# Spider Silk for Tissue Engineering Applications

**DOI:** 10.3390/molecules25030737

**Published:** 2020-02-08

**Authors:** Sahar Salehi, Kim Koeck, Thomas Scheibel

**Affiliations:** 1 Department for Biomaterials, University of Bayreuth, Prof.-Rüdiger-Bormann-Strasse 1, 95447 Bayreuth, Germanykim.koeck@bm.uni-bayreuth.de (K.K.); 2 The Bayreuth Center for Colloids and Interfaces (BZKG), University of Bayreuth, Universitätsstraße 30, 95447 Bayreuth, Germany; 3 The Bayreuth Center for Molecular Biosciences (BZMB), University of Bayreuth, Universitätsstraße 30, 95447 Bayreuth, Germany; 4 The Bayreuth Materials Center (BayMAT), University of Bayreuth, Universitätsstraße 30, 95447 Bayreuth, Germany; 5 Bavarian Polymer Institute (BPI), University of Bayreuth, Universitätsstraße 30, 95447 Bayreuth, Germany

**Keywords:** tissue engineering, biofabrication, hydrogels, coatings, fibers, recombinant spider silk proteins

## Abstract

Due to its properties, such as biodegradability, low density, excellent biocompatibility and unique mechanics, spider silk has been used as a natural biomaterial for a myriad of applications. First clinical applications of spider silk as suture material go back to the 18th century. Nowadays, since natural production using spiders is limited due to problems with farming spiders, recombinant production of spider silk proteins seems to be the best way to produce material in sufficient quantities. The availability of recombinantly produced spider silk proteins, as well as their good processability has opened the path towards modern biomedical applications. Here, we highlight the research on spider silk-based materials in the field of tissue engineering and summarize various two-dimensional (2D) and three-dimensional (3D) scaffolds made of spider silk. Finally, different applications of spider silk-based materials are reviewed in the field of tissue engineering in vitro and in vivo.

## 1. Introduction

Spider silk is a natural material accessed by humans for millennia for various applications. In ancient times, Greeks and Romans stopped the bleeding of battle wounds by covering them with spider silk poultices, and these poultices were also used as an astringent, a styptic and a febrifuge [1,2].

Female orb-weaving spiders produce up to seven different types of silk using different silk glands and spinnerets located at the posterior end of the spider’s abdomen [3]. These seven types of silk have diverse properties depending on their tasks as a protective shelter, dispersal, prey capture device, or as a dragline which the spider uses as a life-line [1,3,4]. Among these seven different silks, major ampullate (MA) silk, which is produced by the major ampullate glands aka as dragline silk, has been investigated in most detail. MA silk has a very high tensile strength and toughness and is used as the frame and radii of the spider’s orb-web [3].

MA silk is composed of at least two classes of proteins, the virtually proline-free major ampullate spidroin (MaSp) 1 and the proline-rich MaSp2 [5,6]. Besides the differences in proline content, MaSp1 and MaSp2 also differ in terms of hydropathicity, with MaSp1 being often hydrophobic and MaSp2 being more hydrophilic although the hydropathy varies between different spider species [7]. In addition to the two long-known spidroin types, a third MaSp3 type was recently identified by Collin et al. [8] and isolated from *Argiope argentata* and *Latrodectus hesperus*. MaSp3 is distinguished from other major ampullate spidroins as it lacks polyalanine and glycine-proline-glycine motifs typically present in MaSp1 and MaSp2. It was shown that MaSp3 repetitive regions contain larger and more polar amino acids than that in MaSp1 or MaSp2 [9] Most spidroins consist of a repetitive core domain flanked by nonrepetitive amino- and carboxyl-terminal domains [10,11] The major ampullate spidroins assemble and form distinguishable substructures, which finally result in a hierarchically structured fiber. This fiber is then in some cases surrounded by glycoproteins and lipids [12].

## 2. Recombinant Spider Silk Production

**Figure 1:**
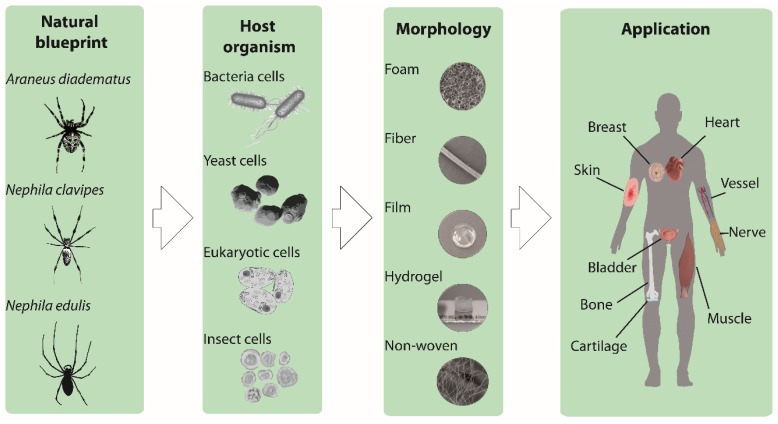
Schematic illustration showing the development of the recombinant spider silk-based biomaterials. Natural spider silk serves as a blue print for recombinant spider silk production. Different production hosts (bacteria, yeast, eukaryotic and insect cells) are shown, as well as various spider silk morphologies (foam, fiber, film, hydrogel, and non-woven mesh) and finally potential applications in tissue engineering.

Harvesting spider silk from natural sources is not easy and does not provide substantial quantities. Biotechnological production enabled the availability of recombinant spider silk proteins in larger quantities and in more consistent quality. Some recombinantly produced spider silk variants are even available commercially [13]. The most used recombinant spider silk proteins are based on sequences from *Nephila clavipes* or *Araneus diadematus* (Figure 1) [14,15].

Generally, the recombinant production of a protein includes the following steps: (1) Natural DNA sequence determination; (2) recombinant DNA design; (3) vector cloning; (4) host organism engineering and transformation; (5) culturing/protein production; and finally (6) protein purification. Different host organisms have already been used to produce spider silk proteins, such as bacteria (e.g., *Escherichia coli*), yeasts (e.g., *Pichia pastoris*), mammalian cells (e.g., hamster kidney cells) or insect cells (Sf9 cells from the fall armyworm *Spodoptera frugiperda*) (Figure 1) [16,17]. The most often used production host for spider silk proteins is *E. coli*, due to fast growth kinetics, high cell density, and easy transformation [18]. One of the main issues concerning recombinant protein production is the discrepancy of the codon usage of spiders and that of the host organism. Additionally, bacterial hosts display another problem in terms of recombinant production, as they often remove repetitive sequences by a mechanism of homologue recombination [19]. Therefore, to be successful, the optimization of the genetic information was necessary using engineered, double-stranded DNA oligonucleotides with adjusted bacterial codon usage [16]. Based on this approach, Huemmerich et al. [7] were able to produce MaSp2 spider silk proteins based on *Araneus diadematus* sequences (ADF3 and ADF4) at sufficient yields using *E. coli* as a bacterial host. Based thereon, several engineered variants have been designed with varying length/ number of core repeats and presence/absence of amino and/or carboxyl-terminal assembly-control domains [17,20,21]. A more detailed view on natural and synthetic spider silk genes can be found in relevant references [7,17].

Biotechnological spider silk production also enables chemical and genetic modifications to tune the properties of the spider silk proteins [22,23]. Modifications can be obtained by manipulation of the amino acid sequence, by incorporation of functional peptides, such as the well-known RGD (arginine, glycine, and aspartic acid) motif from fibronectin for enhanced cell-silk interactions, or amino acids providing functional groups, such as cysteine residues with side groups for subsequent chemical modification of the silk protein. Leal-Egana et al. [24] showed that negatively charged eADF4(C16) is not favorable for cell attachment; however, this cell attachment can be improved by functionalization of the protein with different cell-binding peptides, such as RGD, Ile-Lys-Val-Ala-Val (IKVAV), Tyr-Ile-Gly-Ser-Arg (YIGSR) [25]. Manipulation of the amino acid sequence, for example, by replacing the glutamic acid residue in the repetitive unit of its core domain with lysine ones, yields a positively charged variant of eADF4(C16) [26]. More information about the possibility to modify silk sequences on the genetic level according to the desired application is summarized in specialized reviews on this subject [27,28].

Here, we wanted to focus on the application of spider silk–based materials for tissue regeneration. Tissue regeneration is the process of renewal and regrowth of tissues, and tissue engineering is the selection, design and spatial arrangement of cells and biomaterials for artificial tissue fabrication. Two approaches are typically used in engineering tissues, namely, bottom-up and top-down. Bottom-up is defined as a modular assembly of building units into tissue resembling constructs, and top-down is defined as a simple combination of existing components with a given structure [29]. Regardless of the approach, the “building blocks” to fabricate the tissue-like constructs are biomaterials, cells, and soluble bioactive factors, which are combined at in vitro culturing conditions [30].

A suitable substrate and scaffold for tissue engineering must support cell activities, such as adhesion, migration, proliferation, and differentiation [31,32]. Nowadays, in vitro cell culture studies, such as two-dimensional (2D), as well as three-dimensional (3D) scaffolds, have been evaluated. Mainly hard plastics or glass surface have been used because of ease and convenience. However, tissues comprise an extracellular matrix (ECM) with a porous 3D network structure, and cells are naturally anchored to specific sites on the fibrillary structure of the ECM. Therefore, 3D culture systems are closer to reality, where cells can receive the signals from all three dimensions and show metabolisms and functions close to that of natural tissue. In 3D porous systems, moreover, the high porosity with suitable pore sizes enables the cells to receive nutrition, oxygen, as well as the exchange of waste products [33]. Other properties which have to be considered in the selection of the right substrate for cell culture are biocompatibility, zero toxicity, zero immunogenicity, as well as biodegradation. Furthermore, in tissue engineering applications, degradation of materials is necessary at a rate that allows the formation of new natural tissue; which on the one hand preserves the integrity of the tissue and on the other hand provides space for the native tissue to grow [34]. Considering the type of tissue to be engineered, the mechanical properties of the scaffold also have to be taken into account. For instance, for natural soft tissues the Young’s modulus is in the range of 1–15 kPa, while for hard tissue, such as cancellous bone it is ~350 MPa and for cortical bone ~17 GPa [35]. The matrix stiffness effectively manipulates the cell interaction and regulates cell signaling, migration, differentiation, and motility of cells [36,37,38]. Many of the criteria mentioned above have been shown to be present in spider silk-based 2D and 3D scaffolds, including films, fibers, foams and hydrogels (Figure 1) [25,39].

## 3. One and Two-Dimensional (2D) Spider Silk Materials in Biomedical Applications

### 3.1. Fibers (One Dimensional)

Spider silk fibers are particularly interesting in medical applications, due to their biocompatibility, environmental and mechanical stability as well as a high surface-to-volume ratio [40]. Dragline silk from female adult *Nephila* spiders has been investigated for its potential use as suture threats in the treatment of tendon ruptures. As the success rate of regeneration of flexor tendon injuries is low, sutures are often used for repair. However, one issue of application of sutures is the material, which often causes infection and foreign body reactions. Moreover, the mechanical properties of the sutures are often not compatible with the elasticity and stiffness of the tendon tissue, and over time a reduction in the mechanical stability takes place. Considering the extraordinary mechanical properties of spider silk fibers and the slow degradation rate of the material, Henneck et al. [41] reported stabilizing tendon injuries using spider silk. In this study, the natural spider silk single fibers (from female adult *Nephila* spiders) were braided as a bundle and used as a suture. Various braiding techniques were tested to evaluate the effect of the braiding methods on the tensile properties of the sutures. As a control, conventional surgical sutures were used to compare the properties with that of spider silk. It was found that the braided spider silk showed excellent fatigue behavior and exhibited tensile strengths comparable to those of conventional sutures without any reduction of strength over 1000 fatigue cycles [41].

Almelling et al. and Roloff et al. [42,43] collected dragline silk from *Nephila* species on frames with 20 to 30 fibers in parallel orientation and used this as a scaffold for guiding human model neurons. Neuronal cell bodies contacted the spider silk fibers, and ganglion-like structures were formed within four weeks. Almeling et al. [43] showed Schwann cells (SC) adhered quickly and covered the fibers after cultivation on spider silk fibers. Composite nerve grafts based on acellularized veins, spider silk fibers and SC in matrigel showed migration of cells along the fibers.

Wendt et al. [44] reported the application of spider silk fibers as an innovative matrix for skin repair in plastic reconstructive surgery. Spider dragline silk was harvested directly from *Nephila spp* and was woven on steel frames. After sterilization and culturing of fibroblasts for two weeks, keratinocytes were added to the culture to generate a bilayered skin model. Therefore, layers equivalent to dermis and epidermis were formed. After another three weeks of culture, adhesion of both fibroblasts and keratinocytes on spider silk fibers was observed, while the spider silk fibers guided the growth of the cells.

**Figure 2:**
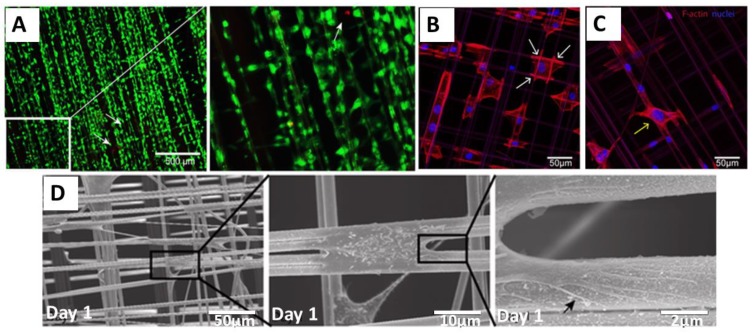
Adhesion and survival of human urothelial cells (HUCs) on natural spider silk fibers (**A**) Live (green) and dead (red) staining of cells cultured on spider silk. Insert: Magnified view of the indicated (white box) area. (**B**,**C**) Confocal images of actin filaments and filopodia development of HUC cells cultured on spider silk meshes after one day of culture showing DAPI (blue) and phalloidin (red)-staining. Attachment sites of cells on fiber meshes are shown with white arrows (**B**), and cells bridging the gap between two fibers are shown with a yellow arrow (**C**). (**D**) Scanning electron microscopy (SEM) image showing cells cultured on spider silk meshes after one day of culture. Consecutive magnifications are shown in sequence. The black arrow points towards the filopodia of cells spread on the fiber surface. Adopted and modified with permission [45]. Copyright 2015, Public Library of Science (PLOS).

Dragline silk from *Nephila edulis* was also tested in pre-clinical models for bladder reconstruction. In this study, the natural spider silk fibers were collected on a stainless-steel frame as a cross-weaved mesh (0.5 × 0.8 cm) and were used for culturing of primary human urothelial cells (HUCs) in vitro without any additional biological coating after autoclaving at 120 °C for an hour. Natural fibers supported adhesion and growth of HUCs, while maintaining their undifferentiated state. Results did not show significant changes in the expression of various epithelial-to-mesenchymal transition, as well as fibrosis-associated genes and only demonstrated a slight reduction in the expression of adhesion-related and cellular differentiation genes [45]. Further, the cell–material interactions with spider silk and the ability of HUCs to form the mucosa of the bladder was studied. Cells could stretch longitudinally alongside the spider silk fibers, and the spider silk extract showed no cytotoxicity (Figure 2). The only issue encountered using the natural fibers was the super-contraction of the fibers. To avoid this, fibers were fixed on the frame for in vitro studies, or the spider silk mesh was sutured into a defect before releasing it from the frame.

Wet spun fibers made of recombinant spider silk proteins have been used in in vivo studies [46]. The miniature spidroin 4RepCT has been released by proteolytic cleavage and spontaneously polymerized into macroscopic fibers. The fibers were formed at the air-liquid interface. 4RepCT and MersilkTM (control) fibers were implanted subcutaneously in rats for seven days. 4RepCT-fibers supported ingrowth of fibroblasts, the formation of new capillaries and were well tolerated.

Wet-spun engineered ADF4(C16) fibers with a diameter of around 44 µm were tested in a rat AV loop model to analyze the angiogenesis and de novo tissue formation of fibrous spider silk. Fibers were produced after dissolving eADF4(C16) in HFIP and wet spinning in 80% 2-Propanol. The spider silk fibrous matrices were filled into Teflon chambers, and the AV loops were placed on top of the spider silk matrices. Further, spider silk fibers were placed on the AV loop before closing the chamber with a lid and fixing it on the thigh musculature of a rat. The results displayed good biocompatibility and vascularization. Even faster biodegradation and a better initiation of vascularization were observed when thinner spider silk fibers were used [47].

### 3.2. Nonwoven Meshes (2D)

Electrospinning [48] has been explored to produce 2D meshes with various fiber orientations, randomly oriented fibers, as well as aligned fibers. Leal-Egana et al. [24] studied the application of electro-spun non-woven scaffolds made of eADF4(C16) as a cell culture substrate. The eADF4(C16) spider silk protein was dissolved in HFIP, and after spinning the fibers were post-treated with methanol vapor in order to convert the α-helical, water-soluble fibers into β-sheet-rich water-stable ones. Interestingly, fibroblasts could adhere to non-woven meshes with fiber diameters of ~900 nm, but not on flat films made of the same protein. As eADF4(C16) lacks specific motifs for cell attachment and cells cannot generate focal adhesions with the surface of this material, fibrous non-woven meshes supported cell adhesion by anchorage of cells on topographical cues. They spread their filopodia and/or lamellipodia between or around individual fibers, explaining the dependency of cell adhesion on the fiber diameter. The best adhesion was achieved on fibers in the range of 700 nm. To avoid any toxicity transferred from the application of toxic organic solvents during electrospinning or post treatment of the silk fibers, a modified system was introduced by DeSimone et al. [49]. They developed an aqueous system to electrospin spider silk proteins, and the authors could show the advantage of this system in supporting the immobilization of biological components in the spinning solution.

### 3.3. Fims and Coatings (2D)

Spider silk films can be prepared by casting, dip- or spray coating or other techniques using solvents like hexofluoro-2-propanol (HFIP), formic acid or aqueous buffers. Evaporation of the solvent leads to a stable silk film which can be peeled off from the surface. The solubility of the resulting films in water is influenced by the secondary structure as determined by Circular Dichroism (CD) and Fourier Transform Infrared (FTIR) Spectroscopy [24,50,51]. Due to the fast evaporation of HFIP as a solvent after the casting process, an equilibrium state of secondary structures was observed with a high amount of α-helical structure. Resulting films required a post-treatment to become water-insoluble. The post-treatment procedure can be carried out with primary alcohols like ethanol or methanol at different percentages ranging from 70% to 100%. [24,39,51] In contrast, films out of formic acid, show an immediate conversion into β-sheet-rich structures resulting in water-insoluble films. [23,50,52,53] Films out of aqueous solutions sometimes require a post-treatment with alcohol [51], and sometimes are further used as cast [51].

Besides water stability, the content of β-sheet structure and crystallinity also influence the mechanical properties of silk films. Increasing β-sheet structure of the film results in increased Young’s modulus and strength, but lowers the elasticity [22,39]. Furthermore, the molecular arrangement of β-sheet structures alters the final mechanical film characteristics. Silk films become stiffer and brittle with a higher number of β-sheet regions and long-range order crystals. Altering the intermolecular interactions by changing the relative humidity of the environment or the addition of plasticizers, such as glycerol, mechanical behavior can be modified [39]. Adding 40% *w/v* glycerol leads to a tenfold increase of the elasticity of eADF4 films, accompanied by a tenfold decrease in Young’s modulus and a slight decrease in strength [52].

The facile preparation of spider silk films makes it a powerful tool for screening the response of cells [22,23,24]. Film characteristics based on surface charge alter the cell attachment [50]. Films made of a positively charged variant of negatively charged eADF4(C16), namely eADF4(κ16), are a good substrate for cardiomyocytes, while cell adhesion on eADF4(C16) films is very poor as depicted above. Interestingly, BALB/3T3 mouse fibroblasts cannot adhere to any of these two films [24,31,54]. With morphological and topographical, as well as genetic modification, e.g. with RGD, the cell adhesion can be improved. Surprisingly the effect of patterned films (width of 20 µm and height of 1 µm) on cell attachment is stronger than the presence of the functional group RGD [53]. In the latter case, it could be shown that after genetic incorporation into spider silk proteins materials made thereof showed similar or slightly better cell adhesion properties than that of chemical modification of the same protein with cyclic RGD [54]. Further, modification of spider silk proteins with IKVAV and YIGSR showed significant improvement of cell interactions by evaluation of the focal adhesions and stress fibers of fibroblast, keratinocytes, endothelial and Schwann cells. In the case of the Schwann cells, strong adherence was shown with spread-out morphology and improved viability [25].

**Figure 3:**
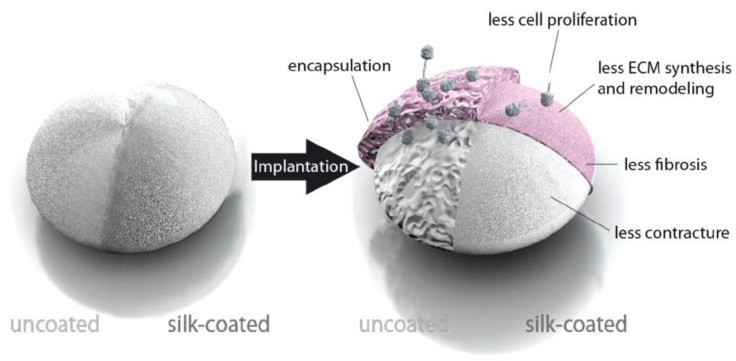
Model of the influence of a spider silk coating on the encapsulation of silicone implants. Adopted and modified with permission [55]. Copyright 2014, John Wiley and Sons.

To modify the interaction of implants with cells, spider silk films can be used as coatings [55,56]. One of the common problems of, e.g., silicone implants is the formation of fibrous tissue when they are implanted in the human body. Therefore, in a study reported by Zeplin et al. [55], breast implants made of silicone were coated with a micrometer thick film of spider silk to improve the surface properties. This film was prepared after dip coating the object into the silk protein solution three times for 120 s followed by a drying interval of 300 s at room temperature. β-sheet formation was induced by treatment of the films in KH_2_PO_4_ (1 M solution) for 120 s and air drying for 120 s. Significant improvement of the implant’s biocompatibility was observed by effectively masking the silicone surface. Coated silicone implants showed significantly reduced capsule thickness, post-operative inflammation, synthesis and remodeling of ECM, and expression of contracture-mediating factors in comparison to uncoated ones (Figure 3) [55]. eADF4(C16) films were also used as a non-adhesive coating on catheters (polyurethane, polytetrafluoroethylene, silicone) as reported by Borkner et al. [57].

## 4. Three-dimensional (3D) Cell Culture System

### 4.1. Porous Foams

Foams with high porosity and interconnected pores can be used as 3D cell culture scaffolds. Cells originally are embedded in a 3D structure made of ECM. In artificial scaffolds, the inner free volume and degradation behavior, as well as the mechanical properties of the employed materials, can be tuned to compare with the natural ECM by controlling the porosity and pore sizes. In a study by Schacht et al. [33] recombinant eADF4(C16) was processed into 3D foams for potential applications in soft tissue engineering. Salt leaching was used to produce highly porous foams. Using various salt crystal sizes, the final pore sizes of the foams were tuned and resulted in various porosities which affected the swelling ratio. The used NaCl crystals did not interfere with the formation of β- sheet structure in the scaffold, and a high porosity above 91% was achieved after dissolving the crystals without further need of additives or cross-linkers. Variation of the protein concentration affected the final mechanical properties and compressive moduli of the foams, which were in the range of soft tissue. RGD motifs present in the variant eADF4(C16)-RGD supported the adhesion and distribution of cells in the foam structure.

Wang et al. [58] reported production of porous foams made of the recombinant spider silk protein pNSR-16. This recombinant spider silk protein also comprises an RGD motif. To fabricate the foams, the spider silk solution in formic acid was mixed with NaCl granules as a porogen, and after evaporating the solvent and dissolving the salt particles, pores in the range of 250 ~ 350 nm remained in the structure. Cytotoxicity was tested using the MTT assay, and no significant differences were shown of cells grown on the scaffolds or treated with their extracts. Hematoxylin and eosin staining showed fibroblasts forming a cell-rich zone on the surface and growing within the structure.

**Figure 4:**
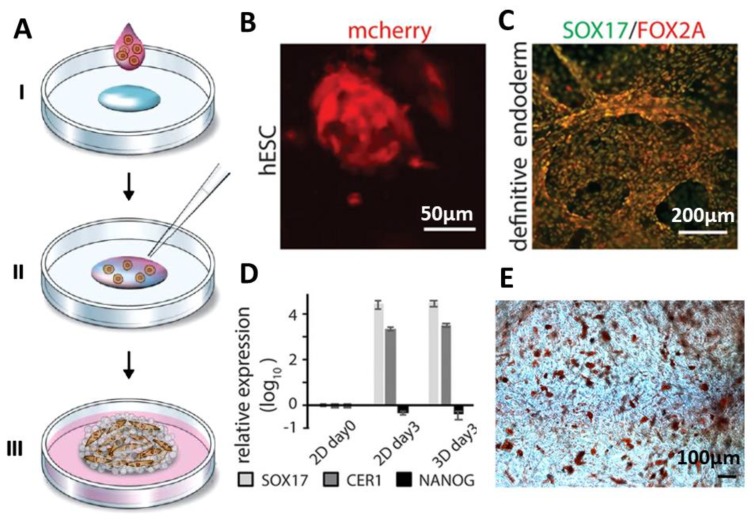
Formation of cell-loaded 3D spider silk foams. (**A**) Foam formation and cell encapsulation. Cells in media mixed with growth medium (pink) are added to a protein solution of fibronectin-silk hybrids (FN-silk) (blue) (I). After gently introducing air bubbles for 5–10 s using a pipette tip (II), the 3D foam containing cells formed. Extra culture medium was added to cover the foam after 30 min (III) [59]. (**B**) Presence of the differentiated human embryonic stem cells (hESC) was visualized by detecting mCherry after 48 h cell integration into the FN-silk foam. (**C**,**D**) Immunostaining visualizing the endodermal markers SOX17 (green) and FOX2A (red) after 3 days of differentiation confirmed the RTqPCR analysis. Expression of genes like SOX17, CER1, NANOG for hESC in an FN-silk foam was compared to that of a 2D culture at day 3 of endodermal induction. Bars represent the mean fold change ± standard deviation (*n* = 4). (**E**) Differentiation of human mesenchymal stem cells (HMSC) in an FN-silk foam into the adipogenic linage, containing lipids were stained by Red Oil (red) (*n* = 2, *n* = 4). Adopted and modified with permission [59]. Copyright 2019, Nature Research.

In a study reported by Johansson et al. [59], a scaffold made of recombinant spider silk protein 4RepCT was genetically modified with a cell adhesion motif from fibronectin (FN) [60]. The scaffold was produced from a silk solution with integrated cells (mouse mesenchymal stem cells) followed by the assembly (Figure 4A). Air bubbles were gently introduced in the solution until an expanding wet foam structure was obtained. Silk assembly into microfibers was promoted by controlling the air-water interface, which enabled homogenous distribution of the cells into the silk material. After three days of culture, cells were well distributed within the 3D network of silk foam. It was shown that adult precursor cells, human embryonic stem cells, could be viably distributed and integrated within the silk foam. Expanding cell aggregates within the 3D foam could be distinguished after 48 h (Figure 4B). After inducing endodermal differentiation, dense layers of cells were formed, which was confirmed using mRNA analysis. (Figure 4C,D). The gene expression has been compared in 2D and 3D samples, and the robust upregulation of SOX17 and CER1 and down regulation of pluripotency (NANOG) was detected (Figure 4D). Differentiation of bone marrow-derived human mesenchymal stem cells (hMSC) into adipogenic lineages was also studied after mixing with the spider silk foam. Johansson et al. [59] showed that lipid droplets were found throughout the scaffolds with integrated cells after expansion and differentiation by adipocyte induction media. (Figure 4E).

Differentiation of adult precursor cells, human skeletal muscle satellite cells (HSkMSC) encapsulated in this foam were also investigated, and after 2 weeks of culture in myogenic differentiation media cells were fully aligned and covered the fibrillar network of the foam.

### 4.2. Hydrogels

**Figure 5:**
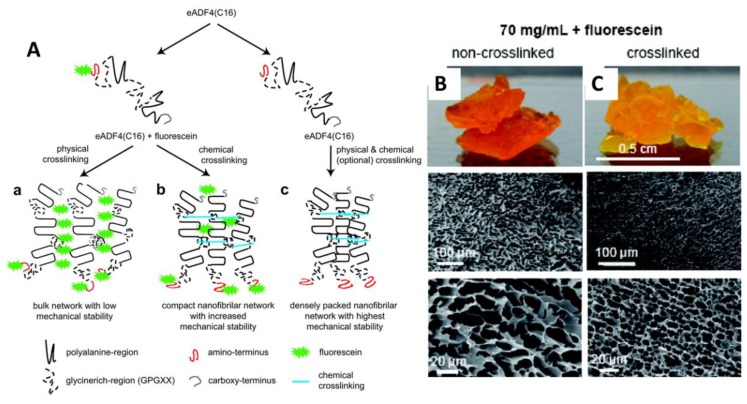
(**A**) Scheme showing the putative mechanism of eADF4(C16) hydrogel formation. (**a**) Chemical modification of spider silk protein with, e.g., fluorescein influences the packing of self-assembling spider silk nanofibrils. (**b**) The nonspecific fluorescein-coupling to tyrosine residues lowers the efficiency of chemical crosslinking of the self-assembled nanofibrils (**c**) In the absence of fluorescein, tightly packed eADF4(C16) hydrogels are formed. Images of the hydrogel and microscopical SEM images of (**B**) non-crosslinked and (**C**) crosslinked eADF4(C16) hydrogels in the presence of 70 mg/mL fluorescein. Adopted and modified with permission [67]. Copyright 2011, American Chemical Society.

Hydrogels are 3D polymer networks with a water content of above 95 % w/w and high swelling rates [61,62]. A variety of naturally and synthetically derived polymers such as agarose, gelatin, chitosan, collagen, alginate, poly(vinyl alcohol) (PVA), and poly- (ethylene oxide) (PEO) have been processed into hydrogels for biomedical applications [63]. In the case of spider silk proteins, self-assembly of ß-sheet-rich nanofibrils through a mechanism of nucleation-aggregation is followed by concentration-dependent gelation [61,64,65,66]). Schacht et al. [67,68] showed hydrogels formation of recombinantly produced spider silk protein eADF4(C16). They have tested various concentrations of the protein, chemical crosslinking, and functionalization with fluorescein to determine the mechanism of hydrogel formation. The most reproducible way to produce eADF4(C16) hydrogels is dialysis at low protein concentrations out of 6 M guanidinium thiocyanate (GdmSCN) into 10 mMTris/HCl, pH 7.5, followed by dialysis against a PEG solution. Based on this procedure, the protein concentration gradually increases and provides time for hydrogel formation, simplifying the handling of the spider silk solution (Figure 5A). Analyzing the morphology of the cross-linked hydrogels showed the formation of highly interconnected sponge-like networks. It was shown that functionalization of the protein with hydrophobic molecules, such as fluorescein strongly influenced assembly and physiochemical properties of the hydrogels, due to interfering with the packing of the nanofibrils during hydrogel formation (Figure 5B,C).

**Figure 6:**
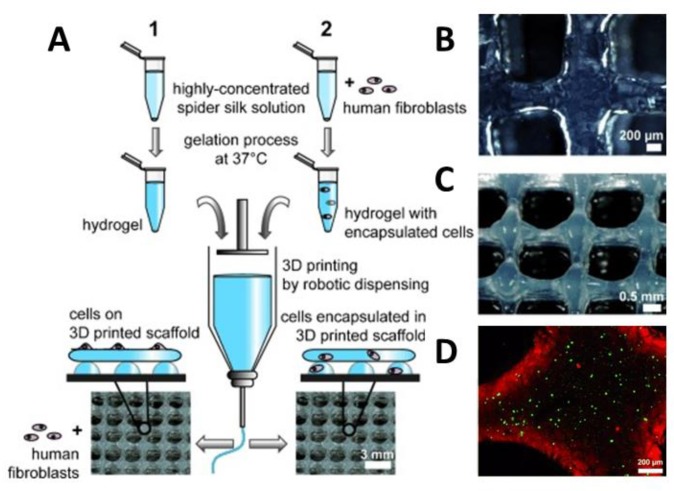
(**A**) Scheme showing the preparation of bioinks containing eADF4(C16) with or without fibroblasts and 3D printing using robotic dispensing. Human fibroblasts were either cultivated on the printed construct (**1**) or encapsulated prior to printing (**2**). Macroscopic images of (**B**) 2-layers of eADF4(C16), (**C**) 8-layer structures and (**D**) confocal laser scanning microscopy of encapsulated cells (live/dead staining) in a printed 2-layer scaffold after 48 h of incubation. Adopted and modified with permission [68]. Copyright 2015, John Wiley and Sons.

One application of spider silk hydrogels is as an ink material in biofabrication, an additive manufacturing technique in which hydrogels are combined with living cells to generate hierarchical tissue-like structures [69]. Development of cytocompatible bioinks, which are cell-friendly and printable, is currently the biggest challenge in the field, and recombinant spider silk hydrogels showed promising properties in this respect. Schacht et al. [67,68] printed eADF4(C16) and eADF4(C16)-RGD hydrogels with fibroblasts by robotic dispensing. The elastic modulus of the 3% (*w/v*) hydrogels with and without RGD motif was 0.2 kPa and 0.02 kPa, respectively. The cytocompatibility and cell adhesion to hydrogels prepared with and without RGD motifs were evaluated using various types of cells, such as fibroblasts, myoblasts, HeLa cells, osteoblasts, and keratinocytes. The cell adhesion of mouse fibroblasts to hydrogels without RGD motifs were weak, and round cells in the form of aggregates were visible. However, in the case of eADF4(C16)-RGD, the cell–material interactions were improved, and mouse fibroblasts were well-spread and showed filopodia formation. Over at least one week of incubation, cells were able to proliferate with high viability (Figure 6A–D). To further improve the cell viability of the spider silk hydrogel, gelatin was used as an additive at a weight-to-weight ratio of 1:200. Gelatin served as a plasticizer and improved the resolution of the printed strands [70].

## 5. Tissue Engineering and Biofabrication Applications

In this section, an overview is provided on various in vitro and in vivo tissue engineering applications of spider silk materials.

### 5.1. Bone and Cartilage Tissue Regeneration

Bone is naturally composed of both inorganic and organic materials being predominately calcium phosphate as an inorganic phase and collagen as an organic substrate. Various biodegradable polymer-based materials have been introduced as components in manufacturing bone replacement implants. Collagen is of particular interest as biomaterial for bone tissue engineering, but it generally suffers from lack of mechanical stability when processed in vitro, and it loses integrity over time [71]. Silks, therefore, have also been in the focus of research, due to their good mechanical properties.

Materials made of recombinant spider silk proteins in the work presented by Hardy et al. [72] and Yang et al. [73] could be biomineralized, and those materials induced an enhanced level of alkaline phosphatase activity of human mesenchymal stem cells which were cultured on the substrates. As the employed spider silk contained multiple carboxylic acid moieties, the calcium ions were able to bind and facilitate the mineralization. When the composite polymer solution of eADF4(C16) and poly(butylene terephthalate) (PBT) or poly(butylene terephthalate-co-poly(alkylene glycol) terephthalate) (PBTAT) was processed into films, calcium carbonate was preferentially deposited on eADF4(C16), but not on the synthetic polymer phase.

Gomes et al. [74] further showed the potential of spider silk proteins for bone regeneration. They used a major ampullate spidroin (MaSp1) of *Nephila clavipes* functionalized with bone sialoprotein (BSP) fusion protein to induce cell attachment, differentiation and deposition of calcium phosphate on the surface of a film yielding an accelerated calcification in vitro after 6 h at 37 °C. Films made of this fusion protein not only induced the deposition of CaP, but also allowed good adhesion of human mesenchymal stem cells and significant improvement of their differentiation. To address the enhancement of biomineralization of the interface of protein-based biomaterials and tissues, the same research group reported the production of silk-hybrids with silica binding peptides [75], as well as genetically engineered spider silk proteins (silk- VTKHLNQISQSY (VTK) fusion proteins) with specific domains (SGRGGLGGQG AGAAAAAGGA GQGGYGGLGSQGT)_15_ and films made thereof [76]. Plowright et al. [75] showed that fusion of spider silk with silica-binding peptide R5 (SSKKSGSYSGSKGSKRRIL), taken from silaffin of *Cerithiopsis fusiformis,* at both termini of the silk sequence (SGRGGLGGQGAGAAAAAGGAGQGGYGGLGSQGT)_15_, derived from the consensus repeat of *Nephila clavipes* MaSp1, induced biosilicification. In this case, the addition of the silica domain to the carboxyl-terminal end showed higher and more controlled silica precipitation. This modified silk also significantly promoted the differentiation of bone marrow hMSCs.

In a different study, Dinjaski et al. fused the domain VTK, which is responsible for the deposition of hydroxyapatite, to spider silk to enhance osteogenesis [76]. The effect of the VTK domain, upon addition to the amino-, carboxyl-terminal or to both termini of the spider silk, was studied concerning material and mineralization properties. The characterization showed that, similar to the previous study [75], the addition of the VTK domain to the carboxyl-terminus of the silk induced more biomineralization. After binding the VTK peptide to the carboxyl-terminus of the silk, more hydroxyl groups were available to interact with mineralizing ions. This effect was combined with a higher flexibility of the hydroxyl-rich part of the peptide leading to a significant effect on mineralization. It was noted that VTK functionalization did not affect the physical properties of the spider silk material. The growth rate and proliferation of hMSCs cultivated on films, cast from this protein showed 3-fold increased osteoinductive properties compared to materials made of spider silk alone.

Tuning differentiation of osteoblasts was examined by Morgan et al. [77] after blending engineered spidroin (originating from *Nephila clavipes* MaSp1) and natural fibroin from silkworm to increase the formation of β-sheets. β-sheet crystalline domains in the produced films guided osteoblastic differentiation of MC3T3-E1 cells. Moreover, the presence of RGD domains supported the adhesion of pre-osteoblasts for improved function.

A preliminary study on the interaction of spider silk materials with chondrocytes was reported by Scheller et al. [78]. Spider silk-elastin fusions were produced in transgenic tobacco, and potato plants, and films made thereof were tested in contact with chondrocytes to study cell adhesion. Chondrocytes showed similar growth rates and faster proliferation on such surfaces in comparison to untreated polystyrene plates. Gellynck et al. [79] studied scaffolds made of various types of silk, including spider egg sac and spider dragline silk fibers concerning the attachment of chondrocytes. Human articular cartilage cells were cultured after steam-sterilizing the fibrous scaffolds, and new ECM was detected on the scaffold after six weeks of culture. Cells were dispersed within the porous structure of the scaffolds and remained attached in the larger pores to the sides and could not fill the pore. However, the smaller pores (100–200µm) were filled with ECM. Production of collagen Type I and II and aggrecan were detected, although the content of collagen type II and aggrecan was higher than that of collagen type I.

### 5.2. Heart Muscle Regeneration

**Figure 7:**
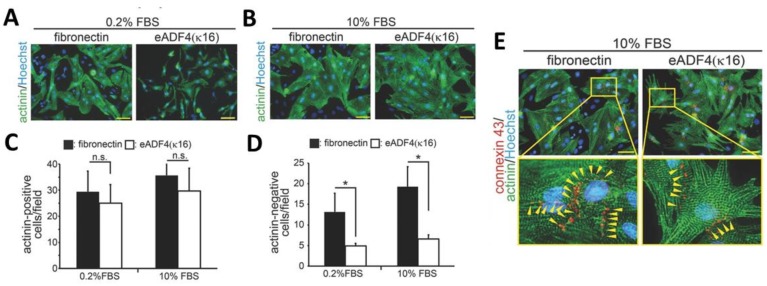
Fluorescent microscopy images of various cell types cultured on eADF4(κ16) films or glass coated with fibronectin after 3 and 48 h. (**A**,**B**) Cardiomyocytes after 48 h of culture on the indicated matrices in the presence of 0.2% (**A**) or 10% FBS (**B**) were stained with sarcomeric-α-actinin (actinin, green) and Hoechst 33342 (nuclei, blue). The expression of sarcomeric-α-actinin in cardiomyocytes and nonmyocytes was analyzed and is presented in (**C**) and (**D**). Data are mean ± SD (*n* = 4), *: *p* < 0.05. n.s.: statistically not significant. Scale bars: 50 µm. (**D**) Cardiomyocytes cultured on eADF4(κ16) films and glass coated with fibronectin (stimulated with 10% FBS) were also stained for connexin 43 (red), sarcomeric-α-actinin (green) and DNA (Hoechst 33342, nuclei, blue). The cardiomyocytes on spider silk films expressed the tight junction marker (connexin 43, yellow arrowheads) indicating cell-to-cell communication. Adopted and modified with permission [50]. Copyright 2017, John Wiley and Sons.

In the work presented by Petzold et al. [23,50] the interaction of primary rat heart cells with films made of eADF4(κ16) was studied. Cardiomyocytes showed good interaction in contact with the positively charged spider silk surface, which showed an interesting selectivity in cell-binding, since nonmyocytes, such as fibroblasts, endothelial cells, and smooth muscle cells did not bind well to such films. Films were prepared upon dip coating of glass coverslips in spider silk solutions in formic acid. To determine the suitability of the spider silk materials for cardiac tissue engineering, the behavior of the most important cell types, such as cardiomyocytes, endothelial cells, fibroblasts, and smooth muscle cells were studied for 3 h, as well as 48 h. Cell type-specific markers were sarcomeric-α-actinin or troponin I (cardiomyocytes), collagen 1 (fibroblasts), smooth muscle actin (smooth muscle cells), and vascular endothelial- (VE-) cadherin (endothelial cells). According to Figure 7 myocytes attached as well, but nonmyocytes attached less efficiently to eADF4(κ16) than to fibronectin films. The behavior of cells on eADF4(κ16) films showed, in contrast to fibronectin films, no hypertrophic effect, but allowed the induction of cardiomyocyte hypertrophy. Cardiomyocytes revealed expression of connexin 43 along cellular junctions, which is responsible for regulating electrical signal propagation between cells (Figure 7D). Moreover, the cardiomyocytes grown on eADF4(κ16) films were able to couple electrically and contracted with the same frequency as on fibronectin films exhibiting no arrhythmia.

### 5.3. Skin Regeneration and Wound Dressings

Baoyong et al. [80] presented the application of recombinant spider silk materials as wound dressings and tested the material in rat models. The feasibility of spider silk porous films/membranes made of pNSR-16 and pNSR-32 (both containing RGD sequences) was tested to cover deep second-degree burn wounds (inflicted with 90 °C boiling water). Collagen was used as a control, and 60 Sprague–Dawley (SD) rats were monitored after 3, 5, 7, 14, and 21 days. The wound-healing rate, histology and the levels of hydroxyproline synthesis were examined. Interestingly, materials made of recombinant spider silk proteins pNSR-16 and pNSR-32 showed much better results than that of the control groups, and higher expression of bFGF was detected on days 7, 14, and 21. Higher content of hydroxyproline (the major amino acid in collagen) in the healed wounds was an indicator of good regeneration of skin in the treated groups.

**Figure 8:**
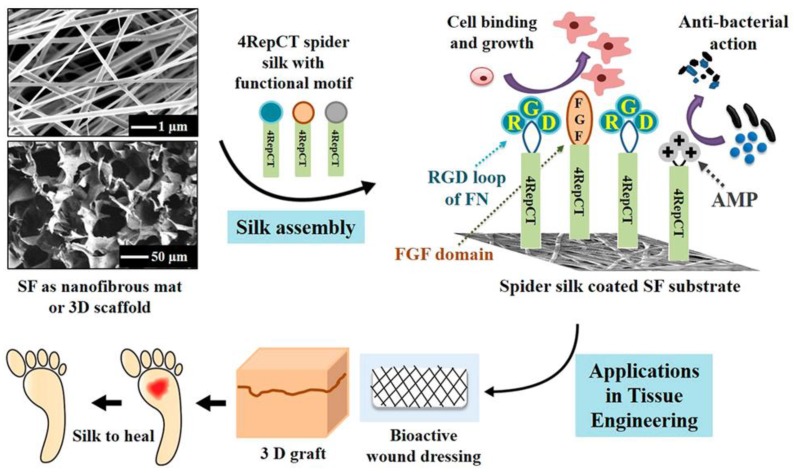
Schematic representation showing the development of functionalized silk substrates for wound healing and skin tissue engineering applications. Silkworm silk fibroin (SF) was used as support material covered by spider silk (4RepCT). The spider silk was functionalized with fused binding motifs from fibronectin containing the RGD sequence (Blue), a growth factor (basic fibroblast growth factor, FGF2) (red), or cationic peptides with antimicrobial properties (AMP) (gray), to enhance the cell-binding activity and cellular growth. Adopted and modified with permission [84]. Copyright 2018, American Chemical Society.

In recent studies presented by Chouhan et al. [81] and Liu et al. [82], recombinant spider silk proteins were genetically modified with a motif from fibronectin to promote cell adhesion. Silk fibroin from silkworms was processed into nanofibrous mats and microporous scaffolds, and those were coated with the recombinant spider silk fusion protein (FN-4RepCT (FN-4RC)) (Figure 8). Third-degree burn wounds in a rat model were covered with such functionalized microporous silk scaffolds. After 14 days of treatment, the functionalized acellular scaffolds demonstrated accelerated wound healing when compared to the commercially available DuoDERM dressing patch and untreated wounds. Moreover, histological assessments confirmed the wound healing in animals treated with functionalized silk in addition to initiation of vascularization and re-epithelialization at the site of injury. The higher expression of collagen type I and type III supported the tissue remodeling and advanced stage of healing upon spider silk treatment. Moreover, this scaffold was used to form a skin prototype after co-culturing multiple cell types. In the adult skin, two layers are distinguishable, a stratified epidermis made of keratinocytes and an underlying dermis mainly made of fibroblasts [83]. To mimic such bilayer, three types of cells, including skin-derived human dermal fibroblasts of neonatal origin (HDFn), human keratinocytes HaCaT and Human dermal microvascular endothelial cells (HDMEC) were co-cultured on the FN-4RC-coated SF scaffolds. After seven days of co-culture, the HaCaT promoted keratinization and showed the development of an epidermal layer.

### 5.4. Peripheral Nerve Regeneration

**Figure 9:**
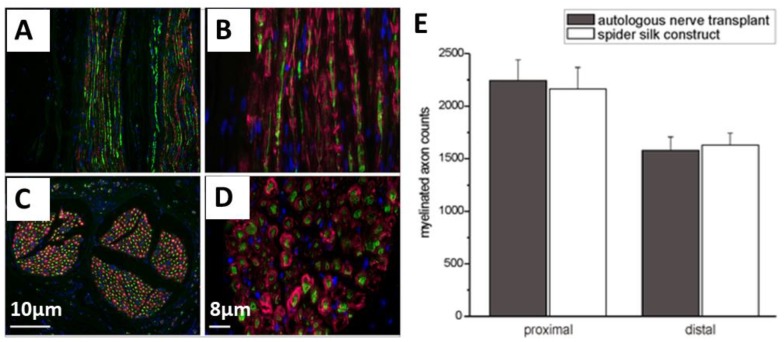
(**A**–**D**) Immunostaining of neurofilaments (green) of regenerated axons and S100 (red) for Schwann cells after transplantation of spider silk constructs in a nerve defect. These images are presenting the regenerated axons and migrated Schwann cells throughout the construct (**C** and **D** showing the cross-sections). Cell nuclei are stained with DAPI (blue) (**E**) Comparison between autologous transplanted nerves and the implanted spider silk construct concerning the number of myelinated axons. Adopted and modified with permission [85]. Copyright 2011, Public Library of Science (PLOS).

One of the therapeutic strategies in promoting axonal regeneration of peripheral nerve injuries is the implantation of biodegradable and biocompatible scaffolds as nerve guidance conduits (NGCs). The main limitation of existing nerve conduits is the gap length, which has to be bridged. Most available NGCs can support bridging up to 30 mm and are designed for small diameter nerves. However, to improve the bridging, tubular structures were introduced filled either with guiding fibers or providing microchannels and encapsulated growth factors. Such an internal morphology is able to guide the axonal extension from the proximal to the distal nerve stumps. In the study of Radtke et al. [85], such constructs were successfully tested in large animal models, and spider silk fibers were tested to bridge the critical size of the nerve defects. They used decellularized vein grafts which were filled with natural spider silk fibers from *Nephila clavipes* as a guiding material to bridge a 6.0 cm model defect in the tibial nerve of adult sheep [42]. The cultured human neurons on the silk fibers showed significant adhesion and migration of cell bodies, as well as differentiation, and extending neurites formed ganglion-like cell structures. The analysis after 6 and 10 months showed the presence of regenerated and myelinated axons throughout the constructs, which is an indicator of the migration of Schwann cells into the constructs. The nodes of Ranvier were defined on regenerated axons in combination with anti-S100 and neurofilaments (Figure 9A–E). The electrophysiological analysis indicated the effectiveness of the fabricated constructs, which was indistinguishable to that of autologous nerve transplants.

**Figure 10:**
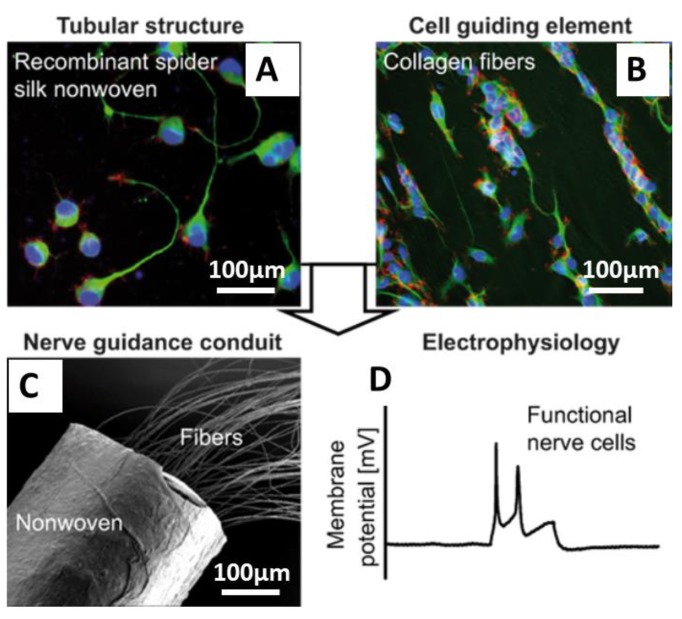
Nerve guidance conduits (NGCs) with cultured NG108-15 cells (**A**,**B**) cells were grown in tubes made of recombinant spider silk nonwoven meshes filled with collagen fibers and differentiated on the collagen fibers into fully functional neurons after three weeks of culture. (**C**) SEM image showing the tubules containing collagen fibers as a cell contact guidance structure. (**D**) Whole-cell patch clamp recordings from cells grown on NGCs, demonstrating the maturation of cells and generation of action potentials in response to current injections (100 pA, 300 ms). Adopted and modified with permission [87]. Copyright 2019, American Chemical Society.

Pawar et al. [86,87] and Aigner et al. [88] reported the development of NGCs using recombinant spider silk eADF4(C16) materials. Pawar et al. [86,87] showed the use of fibers made of eADF4(C16) nonwoven-electrospun meshes filled with microfluidics-produced collagen fibers [87]. In this study, neuroblastoma X glioma hybrid cells NG108-15 were used and differentiated to study the effect of the individual components of the NGC on cell behavior. Cells grown on recombinant spider silk nonwoven meshes and individual collagen fibers successfully differentiated and formed neuronal networks and synapses. This data was confirmed by whole-cell patch clamp recordings (Figure 10). The presence of the nonwoven mesh made of spider silk ensured the structural integrity of the collagen fibers and could prevent the migration of inflammatory cells into the nerve defect. Further, its porous structure will support the nutrient, gas, and waste metabolite exchange in and out of the conduit. The neuronal cells were fully capable of firing action potentials and expressed SNAP-25, which is an indicator for the formation of functional synapses.

Aigner et al. [88] used a different approach and fabricated NGCs based on self-rolling films to allow for encapsulation of PC-12 neuronal cells together with supportive microenvironments. Various inner structures were combined with self-rolling chitosan tubes, such as an anisotropic collagen cryogel with aligned microchannels, aligned spider silk nonwoven meshes and a spider silk film. PC-12 cells cultured in these constructs showed differentiation and formation of aligned neurites within the microchannels, as well as along the aligned fibers of spider silk.

Application of stem cells and neuronal progenitors in contact with recombinant spider silk was studied by Lewicka et al. [89]. In general, this type of cells is capable of differentiating into neurons, astrocytes, and oligodendrocytes. However, access is very limited to the right type of substrate and scaffold, which is supportive for the growth of these cells. Cell proliferation or viability of neuronal stem cells (NSCs) on a recombinant spider silk (4RepCT) matrix and control matrices (conventional poly-L-ornithine and fibronectin (P+F) coated polystyrene plates) did not show any significant differences after 48 h of culture. After stimulating NSCs by bone morphogenetic protein (BMP4) or co-treatment with BMP4 and the signaling factor Wnt3a for seven days (168 h), differentiation of cells was investigated on 4RepCT and controls. BMP4 and BMP4 + Wnt3a stimulation-induced differentiation of cells into functional neuronal cells with pyramidal-like morphology. Results revealed that cells cultured on spider silk surfaces were capable of differentiating into neuronal and astrocytic cells without any significant differences to cells cultured on polystyrene plates with fibronectin coatings. Lewicka et al. [89] also showed that the 3D porous foam structure made of 4RepCT supported NSC attachment, survival and differentiation into astrocytes in 3D. In this study, they cultured NSCs isolated from the cerebral cortices of rat embryos on 3D foams and monitored the interaction of the cells within the porous structure in terms of generation of functional excitatory neurons from NSCs without the addition of animal or human-derived components. NSCs differentiated efficiently into neurons. The functionality of the differentiated neurons was tested after stimulating them with glutamate receptor agonists, such as α-amino-3-hydroxyl-5-methyl-4-isoxazole-propionate (AMPA) receptor, and cells cultured on spider silk foams responded to at least the same extent as control cultures.

### 5.5. Vascularization of Spider Silk Scaffolds

Steiner et al. [47] presented the effective role of recombinant spider silk fibrous scaffolds concerning their vascularization in an arteriovenous fistula (AV loop) model, as mentioned above.

Johansson et al. [59] reported a similar behavior of cells in contact with spider silk foams in a co-culture of endothelial cells and cells from connective tissue. The fraction of endothelial cells in the mixture with mesenchymal stem cells was 2–10%. After two weeks of culture, endothelial cells had gathered and formed millimeter-long branched sprouts. Vessel-like structures could be seen with prominent rings of endothelial cells in the foam, and the diameter of the lumen of these vessels was about 10–20 μm.

## 6. Conclusions

In this review, the potential of spider silk biomaterials has been illustrated for tissue engineering applications. So far, the in vivo study of spider silk-based materials is very limited, although preliminary studies showed that there is no induction of inflammation. When natural fibers or materials made of recombinant spider silk proteins were transplanted in animals, numerous phagocytic cells, consistent with a foreign body reaction were visible after one week of implantation, which is the natural response to any implanted foreign object. However, angiogenesis, which was also detected after one week of transplantation, confirmed that the materials were able to stimulate angiogenesis without amplifying inflammation, which is effective in stimulating wound healing. The degradation of the fibers in one of the studies [46] by macrophages confirmed the biodegradability of the material and showed that the material could be likely replaced by newly grown tissue. Moreover, byproducts of spider silk degradation are nontoxic and can be recognized and removed by the immune system and its responsible cells, which is usually not the case for synthetic polymers like polyesters. The acidic byproducts of synthetic polymers lower the pH, which can negatively affect the viability of cells and surrounding tissues [90]. Another important feature of spider silk-based materials is their slow and gradual rate of degradation. Spider silk materials can remain mechanically stable without becoming brittle or fragile for a significant amount of time at physiological conditions. Slow degradation rates are advantageous in applications, such as in NGCs where the neuronal support can remain for weeks to months, while within the nerve regeneration takes place. However, the degradation can also be adjusted by engineering silk variants with matrix metalloproteinase (MMP) degradation sites [91].

One challenge in the translation of in vitro lab results to clinical in vivo research is the scalability of the techniques and materials. Most of the research and developed tissues or scaffolds might satisfactorily perform in vitro and in very small scales in vivo. However, the applicable engineered tissue in clinical trials has to be often of much bigger sizes.

Although endotoxin-free preparation of recombinant proteins is often challenging [92], complete removal of these byproducts was shown by Wu et al. [93] using purification columns, as well as sterilize silk proteins [83].

It can be stated that unique properties of spider silk materials show some clear advantages, such as low immunogenicity, and biodegradation over currently used ones. Therefore, the more advanced potential applications for spider silk can be seen in the development of artificial grafts for ligaments, nerve, bones, and skin where they possibly can be rapidly used clinically. Towards the future success of spider silk materials, the next step is to initiate further clinical trials in order to obtain regulatory approval for their application in tissue engineering.

## References

[1] Lewis R. (1996). Unraveling the weave of spider silk. BioScience.

[2] Newman J., Newman C. (1995). Oh what a tangled web: The medicinal uses of spider silk. Int. J. Dermatol..

[3] Gosline J.M., DeMont M.E., Denny M.W. (1986). The structure and properties of spider silk. Endeavour.

[4] Stauffer S.L., Coguill S.L., Lewis R.V. (1994). Lewis. Comparison of physical properties of three silks from Nephila clavipes and araneus gemmoides. J. Arachnol..

[5] Liu Y., Sponner A., Porter D., Vollrath F. (2008). Proline and processing of spider silks. Biomacromolecules.

[6] Garb J.E., Haney R.A., Schwager E.E., Gregorič M., Kuntner M., Agnarsson I., Blackledge T.A. (2019). The transcriptome of Darwin’s bark spider silk glands predicts proteins contributing to dragline silk toughness. Commun. Biol..

[7] Huemmerich D., Scheibel T., Vollrath F., Cohen S., Gat U., Ittah S. (2004). Novel assembly properties of recombinant spider dragline silk proteins. Curr. Biol..

[8] Collin M.A., Clarke T.H., Ayoub N.A., Hayashi C.Y. (2018). Genomic perspectives of spider silk genes through target capture sequencing: Conservation of stabilization mechanisms and homology-based structural models of spidroin terminal regions. Int. J. Biol. Macromol..

[9] Kono N., Nakamura H., Ohtoshi R., Moran D.A.P., Shinohara A., Yoshida Y., Fujiwara M., Mori M., Tomita M., Arakawa K. (2019). Orb-weaving spider Araneus ventricosus genome elucidates the spidroin gene catalogue. Sci. Rep..

[10] Eisoldt L., Smith A., Scheibel T. (2011). Decoding the secrets of spider silk. Mater. Today.

[11] Garb J.E., Ayoub N.A., Hayashi C.Y. (2010). Untangling spider silk evolution with spidroin terminal domains. BMC Evol. Biol..

[12] Heim M., Römer L., Scheibel T. (2010). Hierarchical structures made of proteins. The complex architecture of spider webs and their constituent silk proteins. Chem. Soc. Rev..

[13] DeFrancesco L. (2017). Hanging on a thread. Nat. Biotechnol..

[14] Humenik M., Magdeburg M., Scheibel T. (2014). Influence of repeat numbers on self-assembly rates of repetitive recombinant spider silk proteins. J. Struct. Biol..

[15] Dos Santos-Pinto J.R.A., Arcuri H.A., Esteves F.G., Palma M.S., Lubec G. (2018). Spider silk proteome provides insight into the structural characterization of Nephila clavipes flagelliform spidroin. Sci. Rep..

[16] Vendrely C., Scheibel T. (2007). Biotechnological production of spider-silk proteins enables new applications. Macromol. Biosci..

[17] Heidebrecht A., Scheibel T. (2013). Recombinant production of spider silk proteins. Adv. Appl. Microbiol..

[18] Rosano G.L., Ceccarelli E.A. (2014). Recombinant protein expression in Escherichia coli: Advances and challenges. Front. Microbiol..

[19] Arcidiacono S., Mello C., Kaplan D., Cheley S., Bayley H. (1998). Purification and characterization of recombinant spider silk expressed in Escherichia coli. Appl. Microbiol. Biotechnol..

[20] Heidebrecht A., Eisoldt L., Diehl J., Schmidt A., Geffers M., Lang G., Scheibel T. (2015). Biomimetic fibers made of recombinant spidroins with the same toughness as natural spider silk. Adv. Mater. Weinheim..

[21] Eisoldt L. (2013). Funktion und Einfluss der Nicht-Repetitiven, Terminalen Domänen auf Speicherung und Assemblierung von Spinnenseidenproteinen. Ph.D. Thesis.

[22] Humenik M., Smith A.M., Scheibel T. (2011). Recombinant spider silks—Biopolymers with potential for future applications. Polymers.

[23] Aigner T.B., DeSimone E., Scheibel T. (2018). Biomedical applications of recombinant silk-based materials. Adv. Mater. Weinheim..

[24] Leal-Egaña A., Lang G., Mauerer C., Wickinghoff J., Weber M., Geimer S., Scheibel T. (2012). Interactions of fibroblasts with different morphologies made of an engineered spider silk protein. Adv. Eng. Mater..

[25] Widhe M., Johansson U., Hillerdahl C.-O., Hedhammar M. (2013). Recombinant spider silk with cell binding motifs for specific adherence of cells. Biomaterials.

[26] Elena Doblhofer T.S. (2015). Engineering of Recombinant Spider Silk Proteins Allows Defined Uptake and Release of Substances. J. Pharm. Sci..

[27] Saric M., Scheibel T. (2019). Engineering of silk proteins for materials applications. Curr. Opin. Biotechnol..

[28] Herold H.M., Scheibel T. (2017). Applicability of biotechnologically produced insect silks. Z. Nat. C.

[29] Nichol J.W., Khademhosseini A. (2009). Modular tissue engineering: Engineering biological tissues from the bottom up. Soft Matter.

[30] Mason C., Dunnill P. (2008). A brief definition of regenerative medicine. Regen. Med..

[31] Leal-Egaña A., Scheibel T. (2012). Interactions of cells with silk surfaces. J. Mater. Chem..

[32] Janani G., Kumar M., Chouhan D., Moses J.C., Gangrade A., Bhattacharjee S., Mandal B.B. (2019). Insight into silk-based biomaterials: From physicochemical attributes to recent biomedical applications. ACS Appl. Bio Mater..

[33] Schacht K., Vogt J., Scheibel T. (2016). Foams made of engineered recombinant spider silk proteins as 3D scaffolds for cell growth. ACS Biomater. Sci. Eng..

[34] Griffith L.G., Naughton G. (2002). Tissue engineering—Current challenges and expanding opportunities. Science.

[35] Lenhert S., Sesma A., Hirtz M., Chi L., Fuchs H., Wiesmann H.P., Osbourn A.E., Moerschbacher B.M. (2007). Capillary-induced contact guidance. Langmuir.

[36] Wells R.G. (2008). The role of matrix stiffness in regulating cell behavior. Hepatology.

[37] Saez A., Ghibaudo M., Buguin A., Silberzan P., Ladoux B. (2007). Rigidity-driven growth and migration of epithelial cells on microstructured anisotropic substrates. Proc. Natl. Acad. Sci. USA.

[38] Discher D.E., Janmey P., Wang Y.-L. (2005). Tissue cells feel and respond to the stiffness of their substrate. Science.

[39] Spiess K., Lammel A., Scheibel T. (2010). Recombinant spider silk proteins for applications in biomaterials. Macromol. Biosci..

[40] Altman G.H., Diaz F., Jakuba C., Calabro T., Horan R.L., Chen J., Lu H., Richmond J., Kaplan D.L. (2003). Silk-based biomaterials. Biomaterials.

[41] Hennecke K., Redeker J., Kuhbier J.W., Strauss S., Allmeling C., Kasper C., Reimers K., Vogt P.M. (2013). Bundles of spider silk, braided into sutures, resist basic cyclic tests: Potential use for flexor tendon repair. PLoS ONE.

[42] Roloff F., Strauß S., Vogt P.M., Bicker G., Radtke C. (2014). Spider silk as guiding biomaterial for human model neurons. BioMed Res. Int..

[43] Allmeling C., Jokuszies A., Reimers K., Kall S., Vogt P.M. (2006). Use of spider silk fibres as an innovative material in a biocompatible artificial nerve conduit. J. Cell. Mol. Med..

[44] Wendt H., Hillmer A., Reimers K., Kuhbier J.W., Schäfer-Nolte F., Allmeling C., Kasper C., Vogt P.M. (2011). Artificial skin—Culturing of different skin cell lines for generating an artificial skin substitute on cross-weaved spider silk fibres. PLoS ONE.

[45] Steins A., Dik P., Müller W.H., Vervoort S.J., Reimers K., Kuhbier J.W., Vogt P.M., van Apeldoorn A.A., Coffer P.J., Schepers K. (2015). In Vitro evaluation of spider silk meshes as a potential biomaterial for bladder reconstruction. PLoS ONE.

[46] Fredriksson C., Hedhammar M., Feinstein R., Nordling K., Kratz G., Johansson J., Huss F., Rising A. (2009). Tissue response to subcutaneously implanted recombinant spider silk: An In Vivo study. Materials.

[47] Steiner D., Lang G., Fischer L., Winkler S., Fey T., Greil P., Scheibel T., Horch R.E., Arkudas A. (2019). Intrinsic vascularization of recombinant eADF4(C16) spider silk matrices in the arteriovenous loop model. Tissue Eng. Part A.

[48] Doblhofer E., Heidebrecht A., Scheibel T. (2015). To spin or not to spin: Spider silk fibers and more. Appl. Microbiol. Biotechnol..

[49] DeSimone E., Aigner T.B., Humenik M., Lang G., Scheibel T. (2020). Aqueous electrospinning of recombinant spider silk proteins. Mater. Sci. Eng. C Mater. Biol. Appl..

[50] Petzold J., Aigner T.B., Touska F., Zimmermann K., Scheibel T., Engel F.B. (2017). Surface features of recombinant spider silk protein eADF4(κ16)-made materials are well-suited for cardiac tissue engineering. Adv. Funct. Mater..

[51] Spiess K., Ene R., Keenan C.D., Senker J., Kremer F., Scheibel T. (2011). Impact of initial solvent on thermal stability and mechanical properties of recombinant spider silk films. J. Mater. Chem..

[52] Spieß K., Wohlrab S., Scheibel T. (2010). Structural characterization and functionalization of engineered spider silk films. Soft Matter.

[53] Bauer F., Wohlrab S., Scheibel T. (2013). Controllable cell adhesion, growth and orientation on layered silk protein films. Biomater. Sci..

[54] Wohlrab S., Müller S., Schmidt A., Neubauer S., Kessler H., Leal-Egaña A., Scheibel T. (2012). Cell adhesion and proliferation on RGD-modified recombinant spider silk proteins. Biomaterials.

[55] Zeplin P.H., Maksimovikj N.C., Jordan M.C., Nickel J., Lang G., Leimer A.H., Römer L., Scheibel T. (2014). Spider silk coatings as a bioshield to reduce periprosthetic fibrous capsule formation. Adv. Funct. Mater..

[56] Grumezescu A.M., Grumezescu V. (2019). Materials for Biomedical Engineering. Bioactive Materials, Properties, and Applications.

[57] Borkner C.B., Wohlrab S., Möller E., Lang G., Scheibel T. (2016). Surface modification of polymeric biomaterials using recombinant spider silk proteins. ACS Biomater. Sci. Eng..

[58] Wang H.X., Xue Z.X., Wei M.H., Chen D.L., Li M. (2010). A novel scaffold from recombinant spider silk protein in tissue engineering. Advanced Materials Research.

[59] Johansson U., Widhe M., Shalaly N.D., Arregui I.L., Nilebäck L., Tasiopoulos C.P., Åstrand C., Berggren P.-O., Gasser C., Hedhammar M. (2019). Assembly of functionalized silk together with cells to obtain proliferative 3D cultures integrated in a network of ECM-like microfibers. Sci. Rep..

[60] Widhe M., Shalaly N.D., Hedhammar M. (2016). A fibronectin mimetic motif improves integrin mediated cell biding to recombinant spider silk matrices. Biomaterials.

[61] Rammensee S., Huemmerich D., Hermanson K.D., Scheibel T., Bausch A.R. (2006). Rheological characterization of hydrogels formed by recombinantly produced spider silk. Appl. Phys. A.

[62] Yan H., Saiani A., Gough J.E., Miller A.F. (2006). Thermoreversible protein hydrogel as cell scaffold. Biomacromolecules.

[63] Drury J.L., Mooney D.J. (2003). Hydrogels for tissue engineering: Scaffold design variables and applications. Biomaterials.

[64] Kim U.-J., Park J., Li C., Jin H.-J., Valluzzi R., Kaplan D.L. (2004). Structure and properties of silk hydrogels. Biomacromolecules.

[65] Vepari C., Kaplan D.L. (2007). Silk as a biomaterial. Progress Polym. Sci..

[66] Slotta U.K., Rammensee S., Gorb S., Scheibel T. (2008). An engineered spider silk protein forms microspheres. Angew. Chem. Int. Ed Engl..

[67] Schacht K., Scheibel T. (2011). Controlled hydrogel formation of a recombinant spider silk protein. Biomacromolecules.

[68] Schacht K., Jüngst T., Schweinlin M., Ewald A., Groll J., Scheibel T. (2015). Biofabrication of cell-loaded 3D spider silk constructs. Angew. Chem. Int. Ed Engl..

[69] Jungst T., Smolan W., Schacht K., Scheibel T., Groll J. (2016). Strategies and molecular design criteria for 3D printable hydrogels. Chem. Rev..

[70] DeSimone E., Schacht K., Pellert A., Scheibel T. (2017). Recombinant spider silk-based bioinks. Biofabrication.

[71] Riesle J., Hollander A.P., Langer R., Freed L.E., Vunjak-Novakovic G. (1998). Collagen in tissue-engineered cartilage: Types, structure, and crosslinks. J. Cell. Biochem..

[72] Hardy J.G., Torres-Rendon J.G., Leal-Egaña A., Walther A., Schlaad H., Cölfen H., Scheibel T.R. (2016). Biomineralization of engineered spider silk protein-based composite materials for bone tissue engineering. Materials.

[73] Yang L., Hedhammar M., Blom T., Leifer K., Johansson J., Habibovic P., van Blitterswijk C.A. (2010). Biomimetic calcium phosphate coatings on recombinant spider silk fibres. Biomed. Mater..

[74] Gomes S., Leonor I.B., Mano J.F., Reis R.L., Kaplan D.L. (2011). Spider silk-bone sialoprotein fusion proteins for bone tissue engineering. Soft Matter.

[75] Plowright R., Dinjaski N., Zhou S., Belton D.J., Kaplan D.L., Perry C.C. (2016). Influence of silk-silica fusion protein design on silica condensation in vitro and cellular calcification. RSC Adv..

[76] Dinjaski N., Plowright R., Zhou S., Belton D.J., Perry C.C., Kaplan D.L. (2017). Osteoinductive recombinant silk fusion proteins for bone regeneration. Acta Biomater..

[77] Morgan A.W., Roskov K.E., Lin-Gibson S., Kaplan D.L., Becker M.L., Simon C.G. (2008). Characterization and optimization of RGD-containing silk blends to support osteoblastic differentiation. Biomaterials.

[78] Scheller J., Henggeler D., Viviani A., Conrad U. (2004). Purification of spider silk-elastin from transgenic plants and application for human chondrocyte proliferation. Transgenic Res..

[79] Gellynck K., Verdonk P.C.M., van Nimmen E., Almqvist K.F., Gheysens T., Schoukens G., van Langenhove L., Kiekens P., Mertens J., Verbruggen G. (2008). Silkworm and spider silk scaffolds for chondrocyte support. J. Mater. Sci. Mater. Med..

[80] Baoyong L., Jian Z., Denglong C., Min L. (2010). Evaluation of a new type of wound dressing made from recombinant spider silk protein using rat models. Burns.

[81] Chouhan D., Das P., Thatikonda N., Nandi S.K., Hedhammar M., Mandal B.B. (2019). Silkworm silk matrices coated with functionalized spider silk accelerate healing of diabetic wounds. ACS Biomater. Sci. Eng..

[82] Liu X., Miller A.L., Park S., George M.N., Waletzki B.E., Xu H., Terzic A., Lu L. (2019). Two-dimensional black phosphorus and graphene oxide nanosheets synergistically enhance cell proliferation and osteogenesis on 3d printed scaffolds. ACS Appl. Mater. Interfaces.

[83] Li L., Yu Y., Ye G.J., Ge Q., Ou X., Wu H., Feng D., Chen X.H., Zhang Y. (2014). Black phosphorus field-effect transistors. Nat. Nanotechnol..

[84] Chouhan D., Thatikonda N., Nilebäck L., Widhe M., Hedhammar M., Mandal B.B. (2018). Recombinant spider silk functionalized silkworm silk matrices as potential bioactive wound dressings and skin grafts. ACS Appl. Mater. Interfaces.

[85] Radtke C., Allmeling C., Waldmann K.-H., Reimers K., Thies K., Schenk H.C., Hillmer A., Guggenheim M., Brandes G., Vogt P.M. (2011). Spider silk constructs enhance axonal regeneration and remyelination in long nerve defects in sheep. PLoS ONE.

[86] Haynl C., Hofmann E., Pawar K., Förster S., Scheibel T. (2016). Microfluidics-produced collagen fibers show extraordinary mechanical properties. Nano Lett..

[87] Pawar K., Welzel G., Haynl C., Schuster S., Scheibel T. (2019). Recombinant spider silk and collagen-based nerve guidance conduits support neuronal cell differentiation and functionality In Vitro. ACS Appl. Bio Mater..

[88] Aigner T.B., Haynl C., Salehi S., O’Connor A., Scheibel T. (2020). Nerve guidance conduit design based on self-rolling tubes. Mater. Today Bio.

[89] Lewicka M., Rebellato P., Lewicki J., Uhlén P., Rising A., Hermanson O. (2019). Recombinant spider silk protein matrices facilitate multi-analysis of calcium-signaling in neural stem cell-derived AMPA-responsive neurons. BioRxiv.

[90] Naderi H., Matin M.M., Bahrami A.R. (2011). Review paper: Critical issues in tissue engineering: Biomaterials, cell sources, angiogenesis, and drug delivery systems. J. Biomater. Appl..

[91] Müller-Herrmann S., Scheibel T. (2015). Enzymatic degradation of films, particles, and nonwoven meshes made of a recombinant spider silk protein. ACS Biomater. Sci. Eng..

[92] Gorbet M.B., Sefton M.V. (2005). Endotoxin: The uninvited guest. Biomaterials.

[93] Wu S., Johansson J., Damdimopoulou P., Shahsavani M., Falk A., Hovatta O., Rising A. (2014). Spider silk for xeno-free long-term self-renewal and differentiation of human pluripotent stem cells. Biomaterials.

